# Adding dexmedetomidine to morphine-based analgesia reduces early postoperative nausea in patients undergoing gynecological laparoscopic surgery: a randomized controlled trial

**DOI:** 10.1186/s12871-019-0928-y

**Published:** 2020-01-08

**Authors:** Huai Jin Li, Shan Liu, Zhi Yu Geng, Xue Ying Li

**Affiliations:** 1 Department of Anesthesiology, Peking University First Hospital, Beijing, China; 2 Department of Anesthesiology, Beijing Shangdi Hospital, Beijing, China; 3 Department of Biostatics, Peking University First Hospital, Beijing, China

**Keywords:** Dexmedetomidine, Gynecological, Laparoscopic surgery, Patient-controlled analgesia, Postoperative nausea and vomiting

## Abstract

**Background:**

Few studies have investigated the effect of dexmedetomidine on postoperative nausea and vomiting (PONV) in patients underwent gynecological laparoscopic surgery. We investigated if adding dexmedetomidine to a morphine-based patient-controlled analgesia (PCA) could decrease the incidence of PONV in this high-risk patient population.

**Methods:**

In this prospective, randomized, double-blind and placebo-controlled study, 122 patients underwent gynecological laparoscopic surgery were assigned into two groups. Patients in the dexmedetomidine group (Group Dex) received a loading dose of dexmedetomidine 0.4 μg/kg before the end of surgery, followed by morphine 0.5 mg/ml plus dexmedetomidine 1 μg/ml for postoperative i.v. PCA. Patients in the control group (Group Ctrl) received normal saline before the end of surgery, followed by morphine 0.5 mg/ml alone for postoperative i.v. PCA. PCA pump was programmed as followed: bolus dose 2 ml, lockout interval 8 min and background infusion at a rate of 1 ml/h. The primary outcome was the incidence of nausea and vomiting within the first postoperative 24 h.

**Results:**

Although there were no significant differences in regard to the total incidence of PONV (41.0% vs 52.5%, *P* = 0.204), PONV score, time to first onset of PONV, or the need for rescue antiemetics within the first postoperative 24 h between the two groups, the incidence of nausea and total PONV during the first 2 h period was significantly lower in the Group Dex than in the Group Ctrl (9.8% vs 24.6%, *P* = 0.031 and 0.031, respectively). More patients in Group Dex were over sedated or had bradycardia during the PACU compared with Group Ctrl (*P* = 0.040 and 0.036, respectively).

**Conclusion:**

Our protocol in which dexmedetomidine was administered postoperatively – after a loading dose – to intravenous PCA morphine in patients undergoing gynecological laparoscopic surgery, had only early antiemetic effects, while no clinically meaningful antiemetic effect could be evidenced within the first 24 h after surgery.

**Trial registration:**

Current control trial registered at Chictr.org.cn: ChiCTR1800017172. Date registered: 07/16/2018.

## Background

Postoperative nausea and vomiting (PONV) is an unpleasant experience and distressing adverse events after general anesthesia, especially in the first 24 h postoperatively [1].Patients after gynecological surgery are at particularly high risk and the incidence of PONV could even be as high as 80%in this population [2, 3].The Society for Ambulatory Anesthesia consensus guidelines recommended combination antiemetic therapy in high-risk patients population and adoption of prophylactic strategies to reduce the baseline risk of PONV [1]. Opioid-sparing technique is an integral part of enhanced recovery after major gynecological surgery protocol, because it not only reduces PONV but also decreases other opioid-related side effects that can have an influence onpatients’ recovery, such as sedation and postoperative ileus [4].

Dexmedetomidine is a highly selective α2-adrenoreceptor agonist which has sedative, anxiolytic, analgesic, sympatholytic properties and minimal depression of respiratory function. Due to benefits such as maintaining haemodynamic stability, reducing opioid consumption and improving the quality of recovery, it has been widely used in clinical anesthesia, postoperative analgesia and sedation in the intensive care unit [5]. Two meta-analyses demonstrated that intraoperative dexmedetomidine significantly lowered postoperative pain score and opioid consumption, and this could lead to a reduced opioid-related adverse events including PONV [6, 7].

In our previous study, we demonstrated that intraoperative supplemental use of dexmedetomidine resulted in a lower incidence of nausea during the first 2 h postoperatively for patients undergoing gynecological laparoscopic surgery [8]. Intraoperative use of dexmedetomidine was investigated in other other studies about susceptible patients who underwent gynecological laparoscopic surgery [9–12]. Few studies evaluated the effect of postoperative use of dexmedetomidine as patient-controlled analgesia (PCA) regimen in preventing PONV.

A recent study found that adding dexmedetomidine to a fentanyl-based PCA reduced the frequency and severity of postoperative nausea during the time interval 1to 3 h postoperatively in patients underwent lumbar spinal surgery [13].

Thus, in this prospective, randomized, double-blind study, we aimed to evaluate the efficacy of adjunctive dexmedetomidine to morphine-based analgesia for PONV prophylaxis in women undergoin gelective gynecological laparoscopic surgery. Our primary hypothesis was that adding dexmedetomidine to a morphine-based PCA would reduce PONV in this patient population in comparison to only morphine-based PCA.

## Methods

This prospective,randomized, double-blind clinical trial was performed between August 2018 to December 2018 at Peking University First Hospital. The trial was registered at Chictr.org.cn, Number ChiCTR1800017172, on July 162,018, http://www.chictr.org.cn/usercenter.aspx. Ethical approval for this study was provided by the Ethics Committee of Peking University First Hospital, Peking, China (Number 2018–130, principal investigator: Zhi Yu Geng) on 25 July 2018. Written informed content was obtained from all patients before enrollment.

### Participants selection

We used the methodology previously described by our recent study [8].Participant screening was performed the day before surgery. The inclusion criteria were: (1) female patients; (2) age between 18 and 65 years old; (3) scheduled for elective laparoscopic myomectomy or laparoscopy-assisted vaginal hysterectomy. Patients who met any of the following criteria were excluded: (1) American Society of Anesthesiologists physical status >II before surgery, (2) previous history of schizophrenia, Parkinson’s Disease, epilepsy and myasthenia gravis, (3) unable to communicate due to coma, dementia and other diseases, (4) obesity defined as BMI (body mass index) > 30 kg m^− 2^, (5) known sick sinus syndrome, severe bradycardia (heart rate < 50 beats per minute), or severe atrioventricular block without pacemaker before surgery, (6) pre-existing of severe hepatic disease (Child-Pugh class C), (7) pre-existing of chronic renal failure (receive renal replacement therapy preoperatively), (8) Neo-adjuvant chemoradiotherapy before surgery, (9) alcoholism or drug abuse, (10) any regimen of antiemetic, glucocorticoids or psychotropic drugs which are known to have an influence on the occurrence of PONV within 24 h before surgery.

### Randomisation and drug administration

Random numbers were generated by computer software in a 1:1 ratio. Patients were randomized to receive morphine 0.5 mg ml^− 1^ with or without dexmedetomidine 1 μg ml^− 1^. Study drugs were prepared according to the randomization results by a study coordinator. Anesthesiologist and the investigator responsible for the study outcomes assesssment were blinded.

For patients in the dexmedetomidine group (Group Dex), an initial loading dose of 0.4 μg kg^− 1^ dexmedetomidine was given by intravenous infusion 0.5 h before the end of surgery. PCA was begun with 0.5 mg ml^− 1^ morphine plus 1 μg ml^− 1^ dexmedetomidine in 100 ml normal saline. While for patients in the control group (Group Ctrl), normal saline was given 0.5 h before the end of surgery, and PCA was begun with 0.5 mg ml^− 1^ morphine in 100 ml normal saline. For all patients, PCA was programmed to deliver a 2 ml bolus on-demand with a lockout time of 8 min and a background infusion at a rate of 1 ml h^− 1^.

The investigator assessing patients outcomes was blinded to group assignment and blinding was maintained throughout the study period.

### Anesthesia and perioperative care

No pre-medication was administered before induction. Routine monitoring included non-invasive blood pressure, pulse oximetry, electocadiogram, Bispectral index and end-tidal carbon dioxide partial pressure were applied intraoperatively.

All patients received dexamethasone 5 mg before induction. General anesthesia was induced intravenously with 0.03 mg kg^− 1^ midazolam, 2 mg kg^− 1^ propofol, and target controlled infusion of remifentanil with an effect-site concentration of 3 ng ml^− 1^. Rocuronium was administered to facilitate laryngeal mask airway insertion. Total intravenous anesthesia was provided with propofol and remifentanil. Bispectral index was maintained between 40 and 60 during surgery and blood pressure was adjusted within ±20% from baseline. Mechanical ventilation was maintained with a mixture of oxygen and air (FiO_2_ 0.5) and an end-tidal carbon dioxide partial pressure was adjusted between 35 and 55 mmHg intraoperatively. Lactated Ringer’s solution was infused at a rate of 6 ml kg^− 1^ h^− 1^ throughout the surgery.

Morphine 0.1 mg kg^− 1^ and parecoxib sodium 40 mg were administered 0.5 h before the end of surgery. Residual neuromuscular block was reversed with neostigmine (0.04 mg kg^− 1^) and atropine (0.02 mg kg^− 1^) at the end of the surgery.

Upon completion of surgery, laryngeal mask airway was removed and the patient was transferred to the post-anesthesia care unit (PACU) for 1 hour monitoring. The patient-controlled analgesia pump was started and continued until 24 h after surgery.

### Data collection

Data were collected by research personnel who were blinded to the randomization and not involved in the clinical care. The 24 h observation period started at the time of removal of the laryngeal mask airway. The researcher assessed the patients at 2, 6 and 24 h postoperatively. Baseline characteristics of patientssuch as previous history of PONV, chronic smoking, primary risk score for PONV, co-existing systemic diseases and concurrent medication were recorded. Intra-operative parameters including duration of anesthesia and surgery, doses of anesthetics and analgesics, and total fluid administered were collected. Postoperative data including presence and severity of nausea and vomiting, visual analogue scale (VAS) pain scores, the cumulative dose of PCA morphine, requirement for rescue antiemetics, vital signs, sedation scores, and any adverse events were documented.

### Outcome measures

The primary outcome was the incidence of PONV over the first 24 h postoperative hours. Patients who experienced at least one episode of nausea, vomiting or retching or any combination of these during the first 24 h after surgery were considered to have PONV. Patients were asked to rate their degree of nausea using a four-point scale (0 = none, 1 = mild, 2 = moderate, 3 = severe) [14, 15]. Postoperative vomiting was defined as at least one episode of vomiting or retching and the PONV score was rated as 4. Tropisetron 5 mg was used as the rescue antiemetic. If tropisetron failed to relieve the symptom, metoclopramide was administered. Rescue antiemetics were administered on the following conditions: two or more episodes of vomiting or retching, any nausea lasting for more than 30 min, a ‘severe’ degree of nausea or whenever treatment was requested by the patient.

The secondary outcomes included the VAS scores at 2, 6 and 24 h after surgery, the total 24 h morphine consumption, and the occurrence of adverse events during PACU stay. Pain intensity was assessed at PACU, 2 h, 6 h and 24 h postoperatively using an 11-point VAS on which 0 indicated no pain and 10 indicated the worst pain imaginable. In the PACU, supplemental morphine bolus of 2 mg i.v. was administered for moderate pain (VAS ≥4). Sedation levels were assessed using the Ramsay sedation scale (1 = agitated and uncomfortable, 2 = co-operative and orientated, 3 = can follow simple directions, 4 = asleep but strong response to stimulation, 5 = asleep and slow response to stimulation and 6 = asleep and no response to stimulation). Over sedation was defined as a sedation score ≥ 4 [16].Agitation was evaluated using the Ricker sedation-agitation scale and emergence agitation was defined as a sedation-agitation score ≥ 5 [17].

### Sample size calculation

Study sample size was calculated according to our previous studies [3, 8]. In order to detect a 50% reduction in the incidence of PONV (i.e. from 50 to 25%) in the dexmedetomidine group for this patient population, which we considered as clinically meaningful, with a 5% type-I error and power of 80%, we estimated that 55 patients in each group were needed. To allow for a possible dropout rate of 10%, we aimed to enroll 61 patients in each group.

### Statistical analysis

Categorical data are expressed as number (percentage) and were analysed using the *χ* ^2^ test or the Fisher’s exact test as appropriate. Continuous data are expressed as means (standard deviation [SD]) or medians (interquartile range [IQR]) and were analyzed with the unpaired Student’s t-test or Mann–Whitney U test as appropriate. A two-sided *P* value less than 0.05 was considered statistically significant. Statistical analysis was performed using the SPSS 22.0 software (SPSS, Inc., Chicago, Illinois, USA).

## Results

**Fig. 1:**
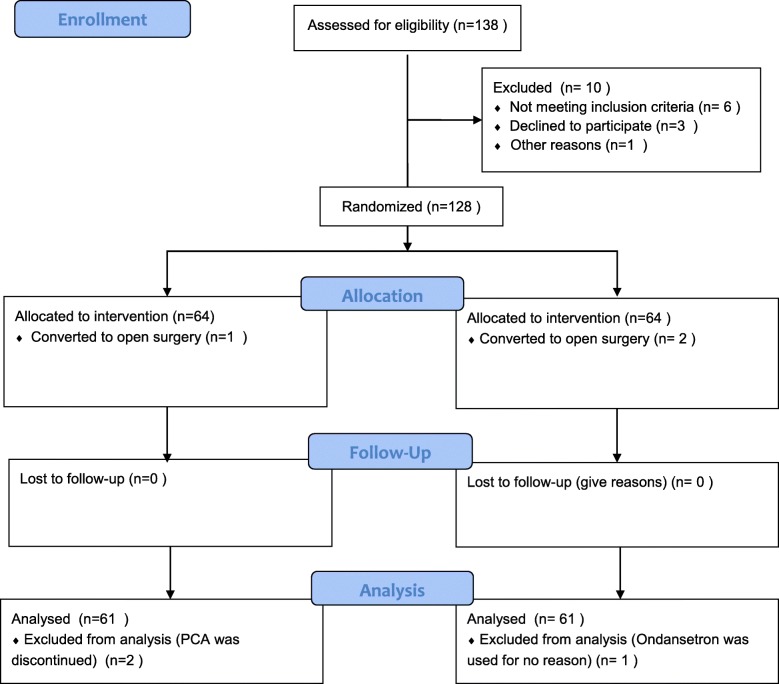
Consort flow diagram

Between August 2018 and December 2018, a total of 128 patients were enrolled and six patients were excluded from the analysis. As a result, 122 patients completed the study: 61 in each group, a flowchart is shown in Fig. 1. There were no significant differences with regard to patient’s baseline characteristics and perioperative data including risk scores of PONV, durations of anesthesia and surgery, propofol and remifentanil doses, and intraoperative fluids between the two groups (Table 1).

**Table 1:** Clinical characteristics and intraoperative variables (*n* = 61)

	Group Dex	Group Ctrl	*P* value
Age (years)	44.0 (8.1)	44.2 (8.4)	0.896
Height (cm)	161.3 (5.8)	160.6 (5.5)	0.503
Weight (kg)	61.0 (8.8)	60.8 (8.8)	0.885
BMI (kgm^−2^)	23.5 (3.1)	23.5 (2.8)	0.872
ASA status I/II (n)	34/27	31/30	0.586
Smoking (n/%)	3 (4.9)	3 (4.9)	1.000
History of motion sickness (n/%)	19 (31.1)	22 (36.1)	0.565
History of PONV (n/%)	4 (6.6)	7 (11.5)	0.343
Apfel score for PONV risk (n/%)			0.492
1	0	0	
2	3 (4.9)	1 (1.6)	
3	38 (62.3)	36 (59.0)	
4	20 (32.8)	24 (39.3)	
Average number of risk scores	3.3 (0.6)	3.4 (0.5)	0.679
Duration of anesthesia(min)	133 (59)	131 (52)	0.837
Duration of surgery(min)	112 (59)	112 (49)	0.969
Intraoperative propofol(mg·kg^− 1^·h^− 1^)	5.4 (1.2)	5.4 (0.8)	0.784
Intraoperative remifentanil (μg·kg^− 1^·h^− 1^)	7.3 (1.4)	7.1 (1.2)	0.367
Intraoperative fluids (ml)	1285 (433)	1239 (417)	0.545

Data are presented as mean (SD) or n (%). *BMI* body mass index

*PONV* postoperative nausea and vomiting

*Dex* dexmedetomidine, *Ctrl*control

The primary outcome was the incidence of PONV over the first 24 h postoperative hours and we found no difference between the two groups (Group Dex vs. Group Ctrl, 25(41.0%) and 32(52.5%), *P* = 0.204) (Table 2).

**Table 2:** Comparison of overall postoperative nausea and vomiting outcomes (*n* = 61)

	Group Dex	Group Ctrl	*P* value
Nausea (n/%)			
0-2 h	6(9.8)	15(24.6)	0.031
2-6 h	7(11.5)	11(18.0)	0.307
6-24 h	21(34.4)	17(27.9)	0.434
Vomiting (n/%)			
0-2 h	3(4.9)	6(9.8)	0.488
2-6 h	3(4.9)	5(8.3)	0.715
6-24 h	8(13.1)	7(11.5)	0.809
PONV(n/%)			
0-2 h	6 (9.8)	15 (24.6)	0.031
2-6 h	7 (11.5)	13 (21.3)	0.142
6-24 h	20 (32.8)	17 (27.9)	0.555
Total 24 h PONV (n/%)	25 (41.0)	32 (52.5)	0.204
PONV score	0 (0, 2.5)	1 (0, 4)	0.226
Time to first PONV (hr)	0(0,4)	0.5(0,3)	0.430
Rescue antiemetics (n/%)	7(11.5)	10(16.4)	0.433

Data are presented as mean (SD), median (lower quartile, upper quartile) or n (%)

*Dex* dexmedetomidine, *Ctrl* control

*PONV* postoperative nausea and vomiting

We found that the incidence of nausea and total PONV during the first 0-2 h after surgery was significantly lower in the Group Dex compared with the Group Ctrl (6(9.8%) and 15(24.6%), *P* = 0.031). We did not find any differences in the incidence of nausea, vomiting and total PONV during the 2-6 h and 6-24 h, PONV score, time to first PONV and the requirement of rescue antiemetics between two groups (Table 2).

The total consumption of morphine during 6-24 h after surgery was significantly less in the Group Dex than in the Group Ctrl (*P* = 0.009), but the cumulative consumption of morphine for the total 24 h after surgery was not significantly different between the groups. The percentage of over sedation and bradycardia during the PACU stay was significantly higher in the Group Dex than in the Group Ctrl (*P* = 0.040 and 0.036, respectively). There were no differences between the two groups regarding VAS score at everytime point, the incidence of shivering, agitation and requiring rescue analgesic in the PACU (Tables 3,4).

**Table 3:** Comparison of PCA morphine consumption and adverse events in PACU (*n* = 61)

	Group Dex	Group Ctrl	*P*value
VAS at PACU	2(1,3)	2(2,3)	0.442
VAS at 2 h	2(1,3)	2(1,3)	0.498
VAS at 6 h	1(0,1)	1(0,1)	0.800
VAS at 24 h	0 (0,1)	0 (0,1)	0.191
Total morphine consumption (mg)			
0-2 h	2.8 (1.5)	2.8 (1.5)	0.907
2-6 h	2.3 (0.6)	2.2 (0.6)	0.280
6-24 h	9.0 (0.1)	9.3 (1.3)	0.009
0-24 h	14.1 (1.8)	14.3 (2.1)	0.524
Use of rescue analgesic (n/%)	10 (16.4)	9(14.8)	0.803

Data are presented as mean (SD) or n (%)

*Dex* dexmedetomidine, *Ctrl* control

*PCA* patient controlled analgesia, *PACU* postanesthesia care unit

**Table 4:** Adverse events in PACU (n = 61)

	Group Dex	Group Ctrl	*P*value
Hypertension (n/%)	2 (3.3)	2 (3.3)	0.611
Hypotension (n/%)	0(0.0)	1 (1.6)	1.000
Respiratory depression (n/%)	0(0.0)	0(0.0)	
Bradycardia (n/%)	6(9.8)	0(0.0)	0.036
Agitation (n/%)	1(1.6)	3(4.9)	0.611
Over sedation (n/%)	10 (16.4)	3 (4.9)	0.040
Shivering (n/%)	7 (11.5)	10 (16.4)	0.433

Data are presented as n (%)

*Dex* dexmedetomidine, *Ctrl* control

*PACU* postanesthesia care unit

## Discussion

Our study showed that for patients who underwent gynecological laparoscopic surgery, adding dexmedetomidine to a morphine-based PCA only decreased the incidence of early nausea during the recovery. There were no significant differences in regard to the total incidence of PONV, time to first onset of PONV, or the need for rescue antiemetics within the first postoperative 24 h between the two groups. Thus, only early antiemetic effects was found when dexmedetomidine was used as an adjunctive analgesic with morphine.

Intraoperative dexmedetomidine administration decreases postoperative pain intensity and opioids consumption compared with placebo. This opioid-sparing effect might lead to a reduction of opioid-related adverse events including PONV [6, 7]. Adding dexmedetomidine to PCA seemed to have some beneficial effects on preventing PONV as well. Du and colleagues [18] used intravenous 0.5 μg kg^− 1^ dexmedetomidine as a loading dose and followed by continuous infusion as an adjunct to butorphanol PCA in patients undergoing total laparoscopic hysterectomy. Their result showed that the administration of dexmedetomidine provided effective analgesia, significant butorphanol sparing and less nausea and vomiting. Another study investigated the effect of dexmedetomidine alone for intravenous PCA after gynecological laparoscopic operation. The result showed that dexmedetomidine alone was effective for postoperative pain control and the incidence of PONV was significant lower in the Dex group [19].

In our present study, we chose the incidence of first 24 h PONV as primary endpoint and demonstrated that dexmedetomidine combined with morphine only reduced early nausea of the first 2 h postoperatively. During the 24 h postoperative period, the cumulative PCA morphine consumption was 14.1 (1.8) mg and 14.3 (2.1) mg respectively in this minimally invasive surgery. In Lin and colleagues’ study [20], they also investigated the effect of combining dexmedetomidine and morphine PCA in patients undergoing total abdominal hysterectomy. Patients receiving dexmedetomidine consumed 29% less PCA morphine and the 4–24 h incidence of nausea was significantly lower in dexmedetomidine group (34% vs 56.3%). Since laparotomy surgery is more painful than laparoscopic surgery, the total morphine consumption and dexmedetomidine dose were much higher than in our study. We speculate this may be the reason for different result.

In our previous research about this PONV susceptible patient population, we found that intraoperative use of dexmedetomidine reduced the incidence of early nausea but not vomiting within 24 h after gynecological laparoscopic surgery [8]. While in this subsequent trial, we focused on the preventive efficacy of postoperative dexmedetomidine when added to morphine PCA and obtained a similar result. The consistent result might be attributed to the initial loading dose of dexmedetomidine before the end of the surgery, since its terminal half-life is about 2 h.

Song and colleagues [13] investigated the effect of combining dexmedetomidine and fentanyl analgesia in patients undergoing lumbar spinal surgery. They found that the Dex group experienced less nausea during 1 to 3 h postoperatively and the intensity of nausea was similar between groups during the first 48 h. Although less PCA fentanyl was required in the Dex groupup to 12 h, there was no statistical significance in the incidence of vomiting between the groups. In addition, in our study, although the cumulative consumption of morphine during 6-24 h after surgery was statistically greater in the Group Ctrl, the incidence of nausea and vomiting was not significantly different between the groups. Since postoperative opioid is one of the primary drivers of PONV, it appears that the intensity of opioid-sparing effect of dexmedetomidine might be crucial to decreasing nausea and vomiting.

The stimulation of nausea and vomiting originates from the inputs of visceral, vestibular and chemoreceptor trigger areas, which are mediated by serotonin, dopamine, histamine and acetylcholine, respectively. Nauseogenic stimulus activates nucleus suchas amugdala, putamen and locus coeruleus, which converted into fear conditioning and emotional triggering. This eventually leads to a strong sensation of nausea [21]. In our study, dexmedetomidine showed some weak and short-lived anti-nausea effect that maybe explained by the properties of α2 agonist. As PONV may be triggered by high catecholamine concentration, it may produce a direct anti-nausea effect through activating the α2-adrenoceptor and decreasing sympathetic tone. Furthermore, dexmedetomidine has concentrate dependent sedative and hypnotic effects that mediated through activation of central α2-receptors in the locus coeruleus. The sedative property mightbe involved in reduing nausea just as benzodiazepines since in our research more patients in the Group Dex were over sedated during the PACU stay.

Dexmedetomidine may increase the risk of postoperative bradycardia [6].When dexmedetomidine combined with sufentanil PCA was used in patient undergoing radical gastrectomy, the incidences of oversedation and bradycardia increased significantly and these side effects were dose-dependent [22]. Despite the low dose regimen of dexmedetomidine in our study, the percentage of patients who experienced bradycardia and over sedation in PACU was significantly higher in the Group Dex. Thus, when determine the optimal dose of dexmedetomidine for postoperative analgesia, the potential increased risk of significant hypotension, bradycardia, respiratory depression and over sedation should be balanced against the maximal beneficial analgesic effect.

There are several limitations of this study. First, in our study PCA was programmed to deliver bolus with a background basal infusion of morphine with or without dexmedetomidine. This continuous infusion dose of morphine might have masked the difference of opioid demands between groups. Second, we only chose one dose and the concentration of dexmedetomidine (1 μg ml^− 1^) was relatively small in PCA. Further dose finding studies of dexmedetomidine are required to confirm the optimal efficacy and safety outcomes for these PONV susceptible patients.

## Conclusions

In conclusion, our protocol in which dexmedetomidine was administered postoperatively – after a loading dose – to intravenous PCA morphine in patients undergoing gynecological laparoscopic surgery, had only early antiemetic effects, while no clinically meaningful antiemetic effect could be evidenced within the first 24 h after surgery.

## Data Availability

The datasets generated and/or analyzed during the current study are not publicly available due to patient confidentiality but are available from the corresponding author on reasonable request.

## Abbreviations

PACU: Post-anesthesia care unit
PCA: Patient-controlled analgesia
PONV: Postoperative nausea and vomiting
VAS: Visual analogue scale
